# Performance improvement for GPS single frequency kinematic relative positioning under poor satellite visibility

**DOI:** 10.1186/s40064-016-2274-6

**Published:** 2016-05-10

**Authors:** Wantong Chen

**Affiliations:** Tianjin Key Lab for Advanced Signal Processing, Civil Aviation University of China, Tianjin, 300300 People’s Republic of China

**Keywords:** Single-frequency GPS, Kinematic relative positioning, Recursive least-squares, Ambiguity fixing, Success rate

## Abstract

Reliable ambiguity resolution in difficult environments such as during setting/rising events of satellites or during limited satellite visibility is a significant challenge for GPS single frequency kinematic relative positioning. Here, a recursive estimation method combining both code and carrier phase measurements was developed that can tolerate recurrent satellite setting/rising and accelerate initialization in motion. We propose an ambiguity dimension expansion method by utilizing the partial ambiguity relevance of previous and current observations. In essence, this method attempts to integrate all useful information into the recursive estimation equation and performs a better least squares adjustment. Using this method, the success rate of the extended ambiguity estimation is independent of the satellite setting and shows robust performance despite poor satellite visibility. Our model allows integration of other useful information into the recursive process. Actual experiments in urban environments demonstrate that the proposed algorithm can improve the reliability and availability of relative positioning.

## Background

With increasing measurement accuracy, single-frequency receivers can serve as primary sensors for relative navigation and positioning in outdoor environments. For this technique, reliable integer ambiguity resolution is required (Tiberius 1998; Zhou et al. 2010; Chen et al. 2015). Incorrect ambiguities can cause position errors of several meters and failed ambiguity resolution reduces function (Parkins 2011). Since the observations from a single epoch do not contain sufficient information to perform reliable ambiguity resolution for single frequency application, the phase and code data from several epochs is required (Hwang and Brown 1990; Jin 1997; Tiberius 1998; Chang et al. 2004). Data processing methods with multi-epoch observations are well documented (Chang and Paige 2003a; Mohiuddin and Psiaki 2007; Giorgi et al. 2010; Buist 2013; Shi et al. 2013; Wang and Lai 2015). These methods assume that the same visible satellites can be observed during at least two consecutive epochs and that the measurements from common satellites and consecutive epochs can be used to achieve ambiguity resolution, a reasonable assumption for most applications given good satellite visibility. However, limited satellite visibility due to the loss of lock may frequently occur due to natural or man-made obstructions such as trees, buildings, bridges, or mountains and this impedes accurate real-time kinematic relative navigation. Additionally, the number of measurement equations may be different for different epochs, leading to different dimensions of the double difference (DD) ambiguities vector. As a consequence, the continuity of the recursive algorithm decreases in schemes that rely on measurements from common satellites of consecutive epochs. Additionally, the information may not be utilized sufficiently, which may prolong its initialization time. To avoid information loss and accelerate initialization, we have developed a new recursive estimator which integrates almost all the available observations for ambiguity resolution. The proposed algorithm can tolerate satellite setting and rising in difficult environments and supports initialization in motion. This is beneficial for some special dynamic applications, which are unable to stop and switch to standby for ambiguity initialization or re-initialization. To achieve reliable ambiguity resolution under poor satellite visibility, we exploited two schemes to strengthen the mathematical model. Firstly, we extended the ambiguity vector by utilizing the partial ambiguity relevance of previous and current observations, and performed a better least squares adjustment. When satellite visibility is limited, it is more reliable to estimate the extended ambiguity vector, since previous observations of invisible satellites may still strengthen the current estimation. Secondly, we designed the algorithm to be efficient, allowing integration of other information to further strengthen the model, such as the integration of the predicted baseline of velocity measurements. We tested the proposed algorithm with actual experiments in urban environments and found improved reliability and availability for kinematic relative positioning.

## The recursive mathematical model

For GPS single frequency relative positioning applications, the LAMBDA method is efficient and optimal (Teunissen 1995, 1999; Verhagen 2004). In this method, continuous carrier phases through enough epochs must be observed to obtain an accurate float solution. The code measurement gives an approximation of the true range between the receiver and the satellite and it would be beneficial to improve the accuracy of the float solution using both the code and the carrier phase measurements. When processing these code measurements with a least squares method, both functional and stochastic models need to be carefully defined (Rizos 1997; Teunissen and Kleusberg 1998). Many realistic modeling approaches are well documented in the literatures (Langley 1997; Tiberius and Kenselaar 2000; Wang et al. 2002; Li et al. 2008; Luo 2013). For simplicity, we developed our algorithm based on the standard GNSS baseline model.

To distinguish the observations of different time points, we utilized the subscript *k* to the *k*th time step, which is referred to as the *k*th epoch. We assume that the proposed method starts at epoch *k*, when the number of common GPS visible satellites *m*_*k*_ exceeds or is equal to 4 and all the lines of sight (LOS) of two antennas to visible satellites have been calculated successfully for both the base station and rover station. The double-difference (DD) baseline model that combines both the code and carrier phase measurements can be written as:

1 $$\begin{aligned}& {\mathbf{y}}_{k} ={\mathbf{ A}}_{k} {\mathbf{a}}_{k} + {\mathbf{B}}_{k} {\mathbf{b}}_{k} + {\varvec{\upmu}}_{k} ,{\varvec{\upmu}}_{k} \sim\;N\left( {0,{\mathbf{Q}}_{{{\mathbf{y}}_{k} }} } \right) \hfill & \\ {\mathbf{a}}_{k} \in Z^{{m_{k} - 1}} ,{\mathbf{b}}_{k} \in R^{3} ,{\mathbf{y}}_{k} \in R^{{2\left( {m_{k} - 1} \right)}} ,{\varvec{\upmu}}_{k} \in R^{{2\left( {m_{k} - 1} \right)}} \hfill &\\ {\mathbf{A}}_{k} \in R^{{2\left( {m_{k} - 1} \right) \times \left( {m_{k} - 1} \right)}} ,{\mathbf{B}}_{k} \in R^{{2\left( {m_{k} - 1} \right) \times 3}} ,{\mathbf{Q}}_{{{\mathbf{y}}_{k} }} \in R^{{2\left( {m_{k} - 1} \right) \times 2\left( {m_{k} - 1} \right)}} \hfill \\ \end{aligned}$$

where ***y*** is the given GNSS data vector, ***µ*** is the noise vector with its variance–covariance (v–c) matrix given by the positive definite matrix ***Q***_***y***_, ***a*** and ***b*** are the integer ambiguity vector and the real-valued baseline vector of order *m*_*k*_ − 1 and 3 respectively, and ***A*** and ***B*** are the given design matrices that link the data vector to the unknown parameters. By applying Cholesky factorization, we decompose the variance–covariance matrix ${\mathbf{Q}}_{{{\mathbf{y}}_{k} }}$ into

2 $${\mathbf{Q}}_{{{\mathbf{y}}_{k} }} = {\mathbf{L}}_{k} \left( {{\mathbf{L}}_{k} } \right)^{\text{T}}$$

Then we calculate the inverse matrix of lower triangular matrix ***L***_*k*_:

3 $${\mathbf{X}}_{k} = \left( {{\mathbf{L}}_{k} } \right)^{ - 1}$$

Multiplying (1) by (3) from the left, we obtain

4 $${\mathbf{X}}_{k} {\mathbf{y}}_{k} {\mathbf{ = X}}_{k} {\mathbf{A}}_{k} {\mathbf{a}}_{k} + {\mathbf{X}}_{k} {\mathbf{B}}_{k} {\mathbf{b}}_{k} + {\mathbf{X}}_{k} {\varvec{\upmu}}_{k}$$

We then define the following notations:

5 $${\bar{\mathbf{y}}}_{k} \equiv {\mathbf{X}}_{k} {\mathbf{y}}_{k} ,{\bar{\mathbf{A}}}_{k} \equiv {\mathbf{X}}_{k} {\mathbf{A}}_{k} ,{\bar{\mathbf{B}}}_{k} \equiv {\mathbf{X}}_{k} {\mathbf{B}}_{k} ,{\bar{\mathbf{\mu }}} \equiv {\mathbf{X}}_{k} {\varvec{\upmu}}_{k}$$

Equation (4) can then be written as

6 $${\bar{\mathbf{y}}}_{k} = \bar{{\mathbf{ A}}}_{k} {\mathbf{a}}_{k} + {\bar{\mathbf{B}}}_{k} {\mathbf{b}}_{k} + {\bar{\mathbf{\mu }}}_{k} ,{\bar{\mathbf{\mu }}}_{k} \sim\;N\left( {0,{\mathbf{I}}_{{m_{k} - 1}} } \right),{\mathbf{a}}_{k} \in Z^{{m_{k} - 1}}$$

Note that the transformed noise vector follows a standard normal distribution. Then let the QR factorization of ${\bar{\mathbf{B}}}_{k}$ be

7 $${\mathbf{Q}}_{k}^{\text{T}} \left( {{\bar{\mathbf{B}}}_{k} } \right)= \left[ {\begin{array}{*{20}c} {{\mathbf{R}}_{{\mathbf{k}}} } \\ {\mathbf{0}} \\ \end{array} } \right], \quad {\mathbf{Q}}_{k}^{\text{T}} \in R^{{\left( {2m_{k} - 2} \right) \times \left( {2m_{k} - 2} \right)}} , \quad {\bar{\mathbf{B}}}_{k} \in R^{{\left( {2m_{k} - 2} \right) \times 3}} , \quad {\mathbf{R}}_{k} \in R^{3 \times 3}$$

where ***Q***_*k*_ is an orthogonal matrix and usually is the product of Householder transformations or Givens rotation matrices (Chang and Paige 2003b), and ***R***_*k*_ is a 3 × 3 nonsingular upper triangular matrix. Since we assume that $m_{k} \ge 4$ so that ${\bar{\mathbf{B}}}_{{\mathbf{k}}}$ almost always has full column rank, we can partition

8 $${\mathbf{Q}}_{k}^{\text{T}} = \left[ {\begin{array}{*{20}c} {{\mathbf{U}}_{k} } \\ {{\mathbf{V}}_{k} } \\ \end{array} } \right], \quad {\mathbf{U}}_{k} \in R^{{3 \times \left( {2m_{k} - 2} \right)}} , \quad {\mathbf{V}}_{k} \in R^{{\left( {2m_{k} - 5} \right) \times \left( {2m_{k} - 2} \right)}}$$

Note that ***U***_*k*_ has the same number of rows as ***R***_*k*_. After the multiplication on the left of (6) by (8), the following equations can be obtained:

9 $$\left[ {\begin{array}{*{20}c} {{\mathbf{U}}_{k} {\bar{\mathbf{y}}}_{k} } \\ {{\mathbf{V}}_{k} {\bar{\mathbf{y}}}_{k} } \\ \end{array} } \right] = \left[ {\begin{array}{*{20}c} {{\mathbf{U}}_{k} {\bar{\mathbf{A}}}_{k} } \\ {{\mathbf{V}}_{k} {\bar{\mathbf{A}}}_{k} } \\ \end{array} } \right]{\mathbf{a}}_{k} + \left[ {\begin{array}{*{20}c} {{\mathbf{R}}_{k} } \\ {\mathbf{0}} \\ \end{array} } \right] \cdot {\mathbf{b}}_{k} + \left[ {\begin{array}{*{20}c} {{\mathbf{U}}_{k} {\bar{\mathbf{\mu }}}_{k} } \\ {{\mathbf{V}}_{k} {\bar{\mathbf{\mu }}}_{k} } \\ \end{array} } \right]$$

Now Eq. (9) can be separated into two parts, and the first one is related to the ambiguity vector and the baseline vector:

10 $${\mathbf{U}}_{k} {\bar{\mathbf{y}}}_{k} = {\mathbf{U}}_{k} {\bar{\mathbf{A}}}_{k} {\mathbf{a}}_{k} + {\mathbf{R}}_{k} {\mathbf{b}}_{k} + {\mathbf{U}}_{k} {\bar{\mathbf{\mu }}}_{k}$$

The second part is only related to the ambiguity vector:

11 $${\mathbf{V}}_{k} {\bar{\mathbf{y}}}_{k} = {\mathbf{V}}_{k} {\bar{\mathbf{A}}}_{k} {\mathbf{a}}_{k} + {\mathbf{V}}_{k} {\bar{\mathbf{\mu }}}_{k}$$

The transformed noise vector follows the same distribution because orthogonal transformation does not change the statistical properties of white noise (Chang and Paige 2003b; Chen and Qin 2012):

12 $$\begin{aligned}& {\mathbf{U}}_{k} {\bar{\mathbf{\mu }}}_{k} \sim\;N\left( {0,{\mathbf{I}}_{3} } \right) \hfill \\ & {\mathbf{V}}_{k} {\bar{\mathbf{\mu }}}_{k} \sim\;N\left( {0,{\mathbf{I}}_{{\left( {2m_{k} - 5} \right)}} } \right) \hfill \\ \end{aligned}$$

For epoch *k* we obtain ${\mathbf{V}}_{k} {\bar{\mathbf{y}}}_{k} \in R^{{\left( {2m_{k} - 5} \right) \times 1}}$ and ${\mathbf{V}}_{k} {\bar{\mathbf{A}}}_{k} \in R^{{\left( {2m_{k} - 5} \right) \times \left( {m_{k} - 1} \right)}}$. Note that the columns of ${\mathbf{V}}_{k} {\bar{\mathbf{A}}}_{k}$ are equal to the order of the ambiguity vector, and less than or equal to the rows of ${\mathbf{V}}_{k} {\bar{\mathbf{A}}}_{k}$, with the assumption that $m_{k} \ge 4$. Next we perform the following orthogonal transformation:

13 $${\mathbf{T}}_{k}^{\text{T}} \left( {{\mathbf{V}}_{k} {\bar{\mathbf{A}}}_{k} } \right) = \left[ {\begin{array}{*{20}c} {{\mathbf{S}}_{k} } \\ {\mathbf{0}} \\ \end{array} } \right], \quad {\mathbf{T}}_{k} \in R^{{\left( {2m_{k} - 5} \right) \times \left( {2m_{k} - 5} \right)}} , \quad {\mathbf{V}}_{k} {\bar{\mathbf{A}}}_{k} \in R^{{\left( {2m_{k} - 5} \right) \times \left( {m_{k} - 1} \right)}} , \quad {\mathbf{S}}_{k} \in R^{{\left( {m_{k} - 1} \right) \times \left( {m_{k} - 1} \right)}}$$

where ***T***_*k*_ is orthogonal and ***S***_*k*_ is nonsingular upper triangular. The orthogonal transformation can be implemented by a sequence of Householder transformations and the zero matrix vanishes for $m_{k} = 4$. Next we divide the orthogonal matrix ***T***_*k*_ into two parts as follows:

14 $${\mathbf{T}}_{k}^{\text{T}} = \left[ {\begin{array}{*{20}c} {{\mathbf{J}}_{k} } \\ {{\mathbf{K}}_{k} } \\ \end{array} } \right],\quad {\mathbf{J}}_{k} \in R^{{\left( {m_{k} - 1} \right) \times \left( {2m_{k} - 5} \right)}} , \quad {\mathbf{K}}_{k} \in R^{{\left( {m_{k} - 4} \right) \times \left( {2m_{k} - 5} \right)}}$$

Multiplying (11) by (14) from the left gives

15 $$\left[ {\begin{array}{*{20}c} {{\mathbf{J}}_{k} \left( {{\mathbf{V}}_{k} {\bar{\mathbf{y}}}_{k} } \right)} \\ {{\mathbf{K}}_{k} \left( {{\mathbf{V}}_{k} {\bar{\mathbf{y}}}_{k} } \right)} \\ \end{array} } \right] = \left[ {\begin{array}{*{20}c} {{\mathbf{S}}_{k} } \\ {\mathbf{0}} \\ \end{array} } \right]{\mathbf{a}}_{k} + \left[ {\begin{array}{*{20}c} {{\mathbf{J}}_{k} \left( {{\mathbf{V}}_{k} {\bar{\mathbf{\mu }}}_{k} } \right)} \\ {{\mathbf{K}}_{k} \left( {{\mathbf{V}}_{k} {\bar{\mathbf{\mu }}}_{k} } \right)} \\ \end{array} } \right]$$

Thus, we can rewrite the top part of (15) as

16 $${\hat{\mathbf{w}}}_{k} = {\mathbf{S}}_{k} {\mathbf{a}}_{k} + {\hat{\mathbf{\mu }}}_{k} ,\quad {\mathbf{S}}_{k} \in R^{{\left( {m_{k} - 1} \right) \times \left( {m_{k} - 1} \right)}} , \quad {\mathbf{a}}_{k} \in R^{{m_{k} - 1}}$$

where ${\hat{\mathbf{w}}}_{k} \equiv {\mathbf{J}}_{k} \left( {{\mathbf{V}}_{k} {\bar{\mathbf{y}}}_{k} } \right),{\hat{\mathbf{\mu }}}_{k} \equiv {\mathbf{J}}_{k} \left( {{\mathbf{V}}_{k} {\bar{\mathbf{\mu }}}_{k} } \right)$. Due to the statistical properties of (12), the transformed noise vector still follows the same distribution:

17 $${\hat{\mathbf{\mu }}}_{k} \sim\,N\left( {0,{\mathbf{I}}_{{m_{k} - 1}} } \right)$$

By solving the upper triangular system (16), the float solution of ambiguity vector at epoch *k* can be estimated as

18 $${\hat{\mathbf{a}}}_{k} = {\mathbf{S}}_{k}^{ - 1} {\hat{\mathbf{w}}}_{k}$$

Obviously we have

19 $${\mathbf{S}}_{k} \left( {{\hat{\mathbf{a}}}_{k} - {\mathbf{a}}_{k} } \right) = {\hat{\mathbf{\mu }}}_{k}$$

Thus, the variance–covariance matrix ${\mathbf{Q}}_{{{\hat{\mathbf{a}}}_{k} }}$ can be written as:

20 $${\mathbf{Q}}_{{{\hat{\mathbf{a}}}_{k} }} = \left( {{\mathbf{S}}_{k}^{\text{T}} {\mathbf{S}}_{k} } \right)^{ - 1}$$

With (18) and (20), the double difference integer ambiguities can be estimated with the well-known LAMBDA method. Once this has been done successfully, the carrier phase data will act as very precise pseudorange data, allowing accurate baseline solution. The conditional least-squares solution can be written as

21 $${\hat{\mathbf{b}}}_{k} \left( {{\mathbf{a}}_{k} } \right) = {\mathbf{R}}_{k}^{ - 1} \left( {{\mathbf{U}}_{k} {\bar{\mathbf{y}}}_{k} - {\mathbf{U}}_{k} {\bar{\mathbf{A}}}_{k} {\mathbf{a}}_{k} } \right)$$

In general, the reliability of ambiguity resolution is described by the probability of correct integer ambiguity estimation or the so-called ambiguity success rate. For the ILS estimator of the unconstrained GNSS baseline model, Teunissen (1999) showed that *P*_*ADOP*_ can be used as an approximation to the ILS success rate, i.e.,

22 $$P_{s,ILS} \approx \;P_{ADOP} = \left( {2\varPhi \left( {\frac{1}{2ADOP}} \right) - 1} \right)^{n}$$

with *n* is the dimension of ambiguity vector and the cumulative normal distribution is

23 $$\varPhi \left( x \right) = \int_{ - \infty }^{x} {\frac{1}{{\sqrt {2\pi } }}} e^{{ - \frac{{v^{2} }}{2}}} dv$$

The ADOP, or Ambiguity Dilution of Precision, is given by $\left| {{\mathbf{Q}}_{{{\hat{\mathbf{a}}}}}^{{}} } \right|^{{\frac{1}{2n}}}$, which is a diagnostic that captures the main characteristics of ambiguity precision (Teunissen and Odijk 1997). When the ambiguities are completely decorrelated, the ADOP equals the geometric mean of the standard deviations of the ambiguities, hence it can be considered as a measure of the average ambiguity precision (Verhagen et al. 2013).The success rate of ambiguity resolution is determined by the strength of the underlying GNSS model; the stronger the model, the higher the success rate (Verhagen 2005; Teunissen 2010; Buist 2013; Chen and Li 2014). In order to improve the strength of the single frequency model, the float solution should be estimated with data from more epochs.

Next, we will deduce the recursive least squares approach based on the baseline model (1) of epoch *k* + 1. The problems of satellite setting and rising are treated with the permutation matrix. This treatment has been well documented in Tiberius (1998) and Chang and Paige (2003b). Here, we employ similar processing in the recursive model, which works very well in our single-frequency case.

At epoch *k* + 1, despite the differences between the baseline vector ${\mathbf{b}}_{k + 1}$ and ${\mathbf{b}}_{k}$, we can obtain the following equation using the same processing approach:

24 $${\mathbf{V}}_{k + 1} {\bar{\mathbf{y}}}_{k + 1} = {\mathbf{V}}_{k + 1} {\bar{\mathbf{A}}}_{k + 1} {\mathbf{a}}_{k + 1} + {\mathbf{V}}_{k + 1} {\bar{\mathbf{\mu }}}_{k + 1}$$

If the tracking is continued without loss of lock for each satellite, the ambiguity vector will remain at current estimation. However, satellite rising/setting often occurs when the satellite is in motion due to obstructions. If we assume that tracking is continued for the reference satellite, we can compare the ambiguities of epoch *k* and partition the ambiguity vector ${\mathbf{a}}_{k + 1}$ into two parts by reordering the elements of the ambiguity vector:

25 $${\mathbf{a}}_{k + 1} = \left[ {\begin{array}{*{20}c} {{\mathbf{a}}_{k + 1}^{rem} } \\ {{\mathbf{a}}_{k + 1}^{new} } \\ \end{array} } \right]$$

where the elements of ${\mathbf{a}}_{k + 1}^{rem}$ correspond to the non-reference satellites which are visible at epoch *k* and remain at epoch *k* + 1, and those of ${\mathbf{a}}_{k + 1}^{new}$ correspond to the non-reference satellites which appear between epochs *k* and *k* + 1. To perform the rearrangement of ambiguities, we construct a permutation matrix $${\mathbf{G}}_{k + 1} = \left[ {\begin{array}{*{20}c} {{\mathbf{G}}_{k + 1}^{rem} } & {{\mathbf{G}}_{k + 1}^{new} } \\ \end{array} } \right]$$ such that

26 $${\mathbf{G}}_{k + 1} {\mathbf{a}}_{k + 1} = \left[ {\begin{array}{*{20}c} {\left( {{\mathbf{G}}_{k + 1}^{rem} } \right)^{\text{T}} {\mathbf{a}}_{k + 1} } \\ {\left( {{\mathbf{G}}_{k + 1}^{new} } \right)^{\text{T}} {\mathbf{a}}_{k + 1} } \\ \end{array} } \right] = \left[ {\begin{array}{*{20}c} {{\mathbf{a}}_{k + 1}^{rem} } \\ {{\mathbf{a}}_{k + 1}^{new} } \\ \end{array} } \right]$$

where ${\mathbf{G}}_{k + 1}^{rem}$ reorders the ambiguity elements of ${\mathbf{a}}_{k + 1}$ to obtain ${\mathbf{a}}_{k + 1}^{rem}$ and ${\mathbf{G}}_{k + 1}^{new}$ reorders the ambiguity elements of ${\mathbf{a}}_{k + 1}$ to obtain ${\mathbf{a}}_{k + 1}^{new}$. Note that the permutation matrix is orthogonal as

27 $${\mathbf{I}} = {\mathbf{G}}_{k + 1} {\mathbf{G}}_{k + 1}^{\text{T}}$$

Using (26) and (27), the following equations can be obtained:

28 $$\begin{aligned} {\mathbf{V}}_{k + 1} {\bar{\mathbf{A}}}_{k + 1} {\mathbf{a}}_{k + 1} &= {\mathbf{V}}_{k + 1} {\bar{\mathbf{A}}}_{k + 1} {\mathbf{G}}_{k + 1} {\mathbf{G}}_{k + 1}^{T} {\mathbf{a}}_{k + 1} \hfill \\ &= \left[ {\begin{array}{*{20}c} {{\mathbf{V}}_{k + 1} {\bar{\mathbf{A}}}_{k + 1} {\mathbf{G}}_{k + 1}^{rem} } & {{\mathbf{V}}_{k + 1} {\bar{\mathbf{A}}}_{k + 1} {\mathbf{G}}_{k + 1}^{new} } \\ \end{array} } \right]\left[ {\begin{array}{*{20}c} {\left( {{\mathbf{G}}_{k + 1}^{rem} } \right)^{T} {\mathbf{a}}_{k + 1} } \\ {\left( {{\mathbf{G}}_{k + 1}^{new} } \right)^{T} {\mathbf{a}}_{k + 1} } \\ \end{array} } \right] \hfill \\ &= \left[ {\begin{array}{*{20}c} {{\mathbf{V}}_{k + 1} {\bar{\mathbf{A}}}_{k + 1} {\mathbf{G}}_{k + 1}^{rem} } & {{\mathbf{V}}_{k + 1} {\bar{\mathbf{A}}}_{k + 1} {\mathbf{G}}_{k + 1}^{new} } \\ \end{array} } \right]\left[ {\begin{array}{*{20}c} {{\mathbf{a}}_{k + 1}^{rem} } \\ {{\mathbf{a}}_{k + 1}^{new} } \\ \end{array} } \right] \hfill \\ \end{aligned}$$

Applying (28) to (24) gives

29 $${\mathbf{V}}_{k + 1} {\bar{\mathbf{y}}}_{k + 1} = \left[ {\begin{array}{*{20}c} {{\mathbf{V}}_{k + 1} {\bar{\mathbf{A}}}_{k + 1} {\mathbf{G}}_{k + 1}^{rem} } & {{\mathbf{V}}_{k + 1} {\bar{\mathbf{A}}}_{k + 1} {\mathbf{G}}_{k + 1}^{new} } \\ \end{array} } \right]\left[ {\begin{array}{*{20}c} {{\mathbf{a}}_{k + 1}^{rem} } \\ {{\mathbf{a}}_{k + 1}^{new} } \\ \end{array} } \right] + {\mathbf{V}}_{k + 1} {\bar{\mathbf{\mu }}}_{k + 1}$$

At this point, we have an equivalent form of (24), which shares common ambiguities with (16), for example, ${\mathbf{a}}_{k + 1}^{rem}$. To integrate (29) into (16) based on the common ambiguities, we use another permutation matrix $${\varvec{\Pi}}_{k + 1} = \left[ {\begin{array}{*{20}c} {{\varvec{\Pi}}_{k + 1}^{his} } & {{\varvec{\Pi}}_{k + 1}^{rem} } \\ \end{array} } \right]$$ such that

30 $${\varvec{\Pi}}_{k + 1}^{T} {\mathbf{a}}_{k} = \left[ {\begin{array}{*{20}c} {\left( {{\varvec{\Pi}}_{k + 1}^{his} } \right)^{T} {\mathbf{a}}_{k} } \\ {\left( {{\varvec{\Pi}}_{k + 1}^{rem} } \right)^{T} {\mathbf{a}}_{k} } \\ \end{array} } \right] = \left[ {\begin{array}{*{20}c} {{\mathbf{a}}_{k + 1}^{his} } \\ {{\mathbf{a}}_{k + 1}^{rem} } \\ \end{array} } \right]$$

where ${\varvec{\Pi}}_{k + 1}^{his}$ reorders the ambiguity elements of ${\mathbf{a}}_{k}$ to obtain ${\mathbf{a}}_{k + 1}^{his}$ and ${\varvec{\Pi}}_{k + 1}^{rem}$ reorders the ambiguity elements of ${\mathbf{a}}_{k}$ to obtain ${\mathbf{a}}_{k + 1}^{rem}$. Here the elements of ${\mathbf{a}}_{k + 1}^{his}$ correspond to the DD integer ambiguity of non-reference satellites that are not visible before epoch *k* + 1. In other words, it includes all the ‘historical’ DD integer ambiguities. Essentially, although some satellites may be invisible during the current epoch, their mathematical equations at previous epoch can be integrated into the current model, since the partial ambiguities remain. By using similar transformations as performed in (27) and (28), the upper triangular system (16) can be written as

31 $$\begin{aligned} {\hat{\mathbf{w}}}_{k} &= {\mathbf{S}}_{k} {\mathbf{a}}_{k} + {\hat{\mathbf{\mu }}}_{k} \hfill \\ &= {\mathbf{S}}_{k} {\varvec{\Pi}}_{k + 1} {\varvec{\Pi}}_{k + 1}^{T} {\mathbf{a}}_{k} + {\hat{\mathbf{\mu }}}_{k} \hfill \\ &= \left[ {\begin{array}{*{20}c} {{\mathbf{S}}_{k} {\varvec{\Pi}}_{k + 1}^{his} } & {{\mathbf{S}}_{k} {\varvec{\Pi}}_{k + 1}^{rem} } \\ \end{array} } \right]\left[ {\begin{array}{*{20}c} {{\mathbf{a}}_{k + 1}^{his} } \\ {{\mathbf{a}}_{k + 1}^{rem} } \\ \end{array} } \right] + {\hat{\mathbf{\mu }}}_{k} \hfill \\ \end{aligned}$$

Then stacking (31) on top of (29) gives

32 $$\left[ {\begin{array}{*{20}c} {{\hat{\mathbf{w}}}_{k} } \\ {{\mathbf{V}}_{k + 1} {\bar{\mathbf{y}}}_{k + 1} } \\ \end{array} } \right] = \left[ {\begin{array}{*{20}c} {{\mathbf{S}}_{k} {\varvec{\Pi}}_{k + 1}^{his} } &\vline & {{\mathbf{S}}_{k} {\varvec{\Pi}}_{k + 1}^{rem} } & {\mathbf{0}} \\ {\mathbf{0}} &\vline & {{\mathbf{V}}_{k + 1} {\bar{\mathbf{A}}}_{k + 1} {\mathbf{G}}_{k + 1}^{rem} } & {{\mathbf{V}}_{k + 1} {\bar{\mathbf{A}}}_{k + 1} {\mathbf{G}}_{k + 1}^{new} } \\ \end{array} } \right]\left[ {\begin{array}{*{20}c} {{\mathbf{a}}_{k + 1}^{his} } \\ \hline {{\mathbf{a}}_{k + 1}^{rem} } \\ {{\mathbf{a}}_{k + 1}^{new} } \\ \end{array} } \right] + \left[ {\begin{array}{*{20}c} {{\hat{\mathbf{\mu }}}_{k} } \\ {{\mathbf{V}}_{k + 1} {\bar{\mathbf{\mu }}}_{k + 1} } \\ \end{array} } \right]$$

This is similar to (13), and we can transform the coefficient matrix of the ambiguity vector to upper triangular form with the following orthogonal transformation:

33 $${\mathbf{P}}_{{_{k + 1} }}^{\text{T}} \left[ {\begin{array}{*{20}c} {{\mathbf{S}}_{k} {\varvec{\Pi}}_{k + 1}^{his} } &\vline & {{\mathbf{S}}_{k} {\varvec{\Pi}}_{k + 1}^{rem} } & {\mathbf{0}} \\ {\mathbf{0}} &\vline & {{\mathbf{V}}_{k + 1} {\bar{\mathbf{A}}}_{k + 1} {\mathbf{G}}_{k + 1}^{{\left( {\mathbf{1}} \right)}} } & {{\mathbf{V}}_{k + 1} {\bar{\mathbf{A}}}_{k + 1} {\mathbf{G}}_{k + 1}^{{\left( {\mathbf{2}} \right)}} } \\ \end{array} } \right] = \left[ {\begin{array}{*{20}c} {{\mathbf{S}}_{k + 1} } \\ {\mathbf{0}} \\ \end{array} } \right]$$

where ${\mathbf{P}}_{{_{k + 1} }}^{{}}$ is orthogonal and ${\mathbf{S}}_{k + 1}$ is nonsingular upper triangular with the same number of rows as the ambiguity vector. Note that this ‘new’ ambiguity vector is dimension-extended and incorporates both the historical and the current DD ambiguities as follows:

34 $${\mathbf{a}}_{k + 1}^{E} = \left[ {\begin{array}{*{20}c} {{\mathbf{a}}_{k + 1}^{his} } \\ \hline {{\mathbf{a}}_{k + 1}^{{}} } \\ \end{array} } \right] = \left[ {\begin{array}{*{20}c} {{\mathbf{a}}_{k + 1}^{his} } \\ \hline {{\mathbf{a}}_{k + 1}^{rem} } \\ {{\mathbf{a}}_{k + 1}^{new} } \\ \end{array} } \right]$$

Multiplying (32) by the orthogonal matrix ${\mathbf{P}}_{{_{k + 1} }}^{\text{T}}$ from the left, we obtain

35 $${\mathbf{P}}_{k + 1}^{\text{T}} \left[ {\begin{array}{*{20}c} {{\hat{\mathbf{w}}}_{k} } \\ {{\mathbf{V}}_{k + 1} {\bar{\mathbf{A}}}_{k + 1} } \\ \end{array} } \right] = \left[ {\begin{array}{*{20}c} {{\mathbf{S}}_{k + 1} } \\ {\mathbf{0}} \\ \end{array} } \right]{\mathbf{a}}_{k + 1}^{E} + {\mathbf{P}}_{k + 1}^{\text{T}} \left[ {\begin{array}{*{20}c} {{\hat{\mathbf{\mu }}}_{k} } \\ {{\mathbf{V}}_{k + 1} {\bar{\mathbf{\mu }}}_{k + 1} } \\ \end{array} } \right]$$

To perform the recursive estimation, we can partition $${\mathbf{P}}_{k + 1}^{\text{T}} = \left[ {\begin{array}{*{20}c} {{\mathbf{C}}_{k + 1}^{\text{T}} } & {{\mathbf{D}}_{k + 1}^{\text{T}} } \\ \end{array} } \right]^{\text{T}}$$ so that the number of rows of ${\mathbf{C}}_{k + 1}$ is equal to the order of ${\mathbf{a}}_{k + 1}^{E}$. Thus, we obtain the following upper triangular system:

36 $${\hat{\mathbf{w}}}_{k + 1} = {\mathbf{S}}_{k + 1} {\mathbf{a}}_{k + 1}^{E} + {\hat{\mathbf{\mu }}}_{k + 1}$$

where $${\hat{\mathbf{w}}}_{k + 1} = {\mathbf{C}}_{k + 1} \left[ {\begin{array}{*{20}c} {{\hat{\mathbf{w}}}_{k} } \\ {{\mathbf{V}}_{k + 1} {\bar{\mathbf{A}}}_{k + 1} } \\ \end{array} } \right]$$ and $${\hat{\mathbf{\mu }}}_{k + 1} = {\mathbf{C}}_{k + 1} \left[ {\begin{array}{*{20}c} {{\hat{\mathbf{\mu }}}_{k} } \\ {{\mathbf{V}}_{k + 1} {\bar{\mathbf{\mu }}}_{k + 1} } \\ \end{array} } \right]$$. Note that Eq. (36) is in similar form as (16) and recursive estimation can be achieved by solving the upper triangular system as

37 $${\hat{\mathbf{a}}}_{k + 1}^{E} = \left( {{\mathbf{S}}_{k + 1} } \right)^{ - 1} {\hat{\mathbf{w}}}_{k + 1}$$

38 $${\mathbf{Q}}_{{{\hat{\mathbf{a}}}_{k + 1}^{E} }} = \left( {{\mathbf{S}}_{k + 1}^{\text{T}} {\mathbf{S}}_{k + 1} } \right)^{ - 1}$$

In this recursive estimator, the ambiguity vector is extended by allowing satellite rising and setting. Once the extended ambiguity vector ${\mathbf{a}}_{k + 1}^{E}$ is resolved, we can extract the current ambiguity vector ${\mathbf{a}}_{k + 1}$ and calculate the current conditional baseline vector in the same way as (21):

39 $${\hat{\mathbf{b}}}_{k + 1} \left( {{\mathbf{a}}_{k + 1} } \right) = {\mathbf{R}}_{k + 1}^{ - 1} \left( {{\mathbf{U}}_{k + 1} {\bar{\mathbf{y}}}_{k + 1} - {\mathbf{U}}_{k + 1} {\bar{\mathbf{A}}}_{k + 1} {\mathbf{a}}_{k + 1} } \right)$$

The recursive process can always be achieved if $m_{k + 1} \ge 4$ and if ${\mathbf{a}}_{k + 1}^{rem}$ is not empty. To more clearly demonstrate the rearrangement of the ambiguities, we have presented a case in Table 1 which shows how the extended ambiguity vector changes as satellite setting/rising occurs. The notation PRN^epoch^ denotes the DD integer ambiguity of the non-reference satellite PRN to the reference satellite with its ‘birth moment’ denoted with the superscript ‘epoch’. Table 1 shows that satellite setting cannot change the number of ambiguities.

**Table 1:** A case for satellite setting/rising and the rearrangement of ambiguity vector

Epoch	Ref. sat	Non-reference satellite list	${\mathbf{a}}_{k}^{his}$	${\mathbf{a}}_{k}^{rem}$	${\mathbf{a}}_{k}^{new}$
*k*	6	2/9/5/17/12/23		2^*k*^/9^*k*^/5^*k*^/17^*k*^/12^*k*^/23^*k*^	
*k* + 1 ~ *k* + 3	6	2/9/5/12/23	17^*k*^	2^*k*^/9^*k*^/5^*k*^/12^*k*^/23^*k*^	
*k* + 4 ~ k + 7	6	2/9/5/12	17^*k*^/23^*k*^	2^*k*^/9^*k*^/5^*k*^/12^*k*^	
*k* + 8	6	2/9/5/12/17	17^*k*^/23^*k*^	2^*k*^/9^*k*^/5^*k*^/12^*k*^	17^*k*+8^
*k* + 9 ~*k* + 13	6	2/9/5/12/17	17^*k*^/23^*k*^	2^*k*^/9^*k*^/5^*k*^/12^*k*^/17^*k*+8^	
*k* + 14	6	2/9/5/12/17/23	17^*k*^/23^*k*^	2^*k*^/9^*k*^/5^*k*^/12^*k*^/17^*k*+8^	23^*k*+14^
*k* + 15 ~*k* + 23	6	2/9/5/12/17/23	17^*k*^/23^*k*^	2^*k*^/9^*k*^/5^*k*^/12^*k*^/17^*k*+8^/23^*k*+14^	
*k* + 24 ~*k* + 28	6	2/9/5/12/23	17^*k*^/23^*k*^/17^*k*+8^	2^*k*^/9^*k*^/5^*k*^/12^*k*^/23^*k*+14^	
*k* + 29	6	2/9/5/23	17^*k*^/23^*k*^/17^*k*+8^/12^*k*^	2^*k*^/9^*k*^/5^*k*^/23^*k*+14^	

From GPS L1 data at a rate of 1 Hz and start epoch *k* = 47,009

## Global and local success rates

For simplicity, the following notations are utilized for the design matrix of (32):

40 $${\mathbf{W}}_{k + 1} \equiv \left[ {\begin{array}{*{20}c} {{\mathbf{S}}_{k} {\varvec{\Pi}}_{k + 1}^{his} } &\vline & {{\mathbf{S}}_{k} {\varvec{\Pi}}_{k + 1}^{rem} } & {\mathbf{0}} \\ {\mathbf{0}} &\vline & {{\mathbf{V}}_{k + 1} {\bar{\mathbf{A}}}_{k + 1} {\mathbf{G}}_{k + 1}^{rem} } & {{\mathbf{V}}_{k + 1} {\bar{\mathbf{A}}}_{k + 1} {\mathbf{G}}_{k + 1}^{new} } \\ \end{array} } \right] \equiv \left[ {\begin{array}{*{20}c} {{\mathbf{H}}^{his} } &\vline & {{\mathbf{H}}^{cur} } \\ \end{array} } \right]$$

Thus the variance–covariance matrix of ${\mathbf{a}}_{k + 1}^{E}$ can be written as

41 $${\mathbf{Q}}_{{{\hat{\mathbf{a}}}_{k + 1}^{E} }} = \left( {{\mathbf{W}}_{k + 1}^{\text{T}} {\mathbf{W}}_{k + 1} } \right)^{ - 1} = \left[ {\begin{array}{*{20}c} {\left( {{\mathbf{H}}^{his} } \right)^{\text{T}} {\mathbf{H}}^{his} } & {\left( {{\mathbf{H}}^{his} } \right)^{\text{T}} {\mathbf{H}}^{cur} } \\ {\left( {{\mathbf{H}}^{cur} } \right)^{\text{T}} {\mathbf{H}}^{his} } & {\left( {{\mathbf{H}}^{cur} } \right)^{\text{T}} {\mathbf{H}}^{cur} } \\ \end{array} } \right]^{ - 1} = \left[ {\begin{array}{*{20}c} {{\mathbf{Q}}_{{{\hat{\mathbf{a}}}_{k + 1}^{his} }} } & {{\mathbf{Q}}_{{{\hat{\mathbf{a}}}_{k + 1}^{his} {\hat{\mathbf{a}}}_{k + 1}^{{}} }} } \\ {{\mathbf{Q}}_{{{\hat{\mathbf{a}}}_{k + 1}^{{}} {\hat{\mathbf{a}}}_{k + 1}^{his} }} } & {{\mathbf{Q}}_{{{\hat{\mathbf{a}}}_{k + 1}^{{}} }} } \\ \end{array} } \right]$$

Based on (22), the success rate of the extended ambiguity vector can be estimated. Since ${\mathbf{a}}_{k + 1}^{E}$ contains all the ambiguity elements, we refer to it as the ‘global success rate’, written as *P*_G_. We need to calculate the conditional baseline vector with ${\mathbf{a}}_{k + 1}$, see (39). To do this, one can partition ${\mathbf{Q}}_{{{\hat{\mathbf{a}}}_{k + 1}^{E} }}$ as (41) or directly calculate the v-c matrix of the partial float ambiguity vector ${\hat{\mathbf{a}}}_{k + 1}$ by applying the well-known partitioned matrix inversion lemma:

42 $${\mathbf{Q}}_{{{\hat{\mathbf{a}}}_{k + 1}^{{}} }} = \left[ {\left( {{\mathbf{H}}^{cur} } \right)^{\text{T}} {\mathbf{H}}^{cur} - \left( {{\mathbf{H}}^{cur} } \right)^{\text{T}} {\mathbf{H}}^{his} \left( {\left( {{\mathbf{H}}^{his} } \right)^{\text{T}} {\mathbf{H}}^{his} } \right)^{ - 1} \left( {{\mathbf{H}}^{his} } \right)^{\text{T}} {\mathbf{H}}^{cur} } \right]^{ - 1}$$

**Fig. 1:**
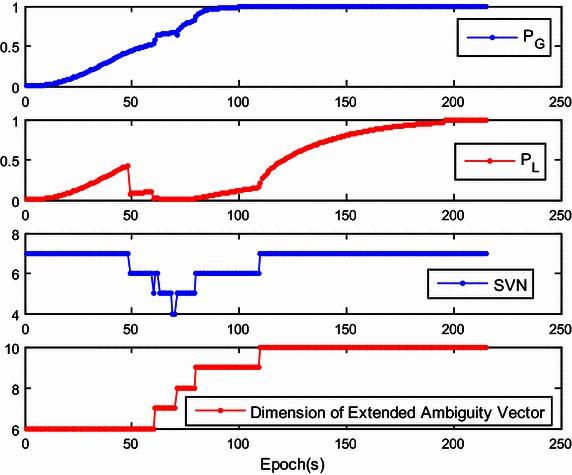
Comparison of global success rate and local success rate for SVN fluctuation

The success rate given by ${\mathbf{Q}}_{{{\hat{\mathbf{a}}}_{k + 1}^{{}} }}$ is referred to as the ‘local success rate’, and is denoted as *P*_L_. If *P*_L_ is much higher than *P*_G_, only the current ambiguity vector can be estimated with ${\hat{\mathbf{a}}}_{k + 1}$ and ${\mathbf{Q}}_{{{\hat{\mathbf{a}}}_{k + 1}^{{}} }}$, regardless of ${\mathbf{a}}_{k + 1}^{his}$. However, *P*_L_ may be sensitive to the number of the visible satellites and will dramatically drop when the number of visible satellites decreases. To illustrate this, Fig. 1 shows the trends of *P*_L_ and *P*_G_ with the number of visible satellites. At the initial stage of the kinematic test, obstructions in the urban environment reduce the number of visible satellites to four. This number gradually rises to seven. Different than *P*_L_, *P*_G_ is almost always growing and finally approaches 1. Therefore, the ambiguity resolution for the extended ambiguity vector is preferred in actual applications.

The dimension of the extended ambiguity vector cannot be reduced by satellite setting. However, the dimension increases as the number of visible satellites increases. Thus, the progressive increase of ambiguities may increases the burden of computation. To deal with this problem during real-time application, a fast integer least-squares estimation is required for high-dimensional ambiguity resolution, which has been described previously (Chang et al. 2005; Zhou 2010; Jazaeri et al. 2012). Another simple approach is to limit the growth of the dimension of the historical DD ambiguity vector ${\mathbf{a}}_{k + 1}^{his}$. For instance, when it exceeds the preset threshold, we can remove the first ambiguity element of the upper triangular system (36), thus reducing the total ambiguity dimension by 1. Here, the threshold was set as 10 and little effect on the global success rate was found.

## Ambiguity validation for ambiguity initialization

The final aim of initialization is to perform a rapid and successful ambiguity resolution for real-time application. Thus, the validation procedure is required for initialization. We used the ratio test, a commonly used validation procedure described in Verhagen (2004) and Teunissen and Verhagen (2009). In this method, the ratio of the squared norm of the ambiguity residuals in the metric of the covariance for the best and second best integer solution, as obtained from LAMBDA, is examined to perform a validation calculation:

43 $$\frac{{\left( {{\hat{\mathbf{a}}}_{k + 1}^{E} - {\tilde{\mathbf{a}}}_{k + 1}^{E} } \right)^{\text{T}} {\mathbf{Q}}_{{{\hat{\mathbf{a}}}_{k + 1}^{E} }}^{ - 1} \left( {{\hat{\mathbf{a}}}_{k + 1}^{E} - {\tilde{\mathbf{a}}}_{k + 1}^{E} } \right)}}{{\left( {{\hat{\mathbf{a}}}_{k + 1}^{E} - {\mathbf{\overset{\lower0.5em\hbox{$\smash{\scriptscriptstyle\smile}$}}{a} }}_{k + 1}^{E} } \right){\mathbf{Q}}_{{{\hat{\mathbf{a}}}_{k + 1}^{E} }}^{ - 1} \left( {{\hat{\mathbf{a}}}_{k + 1}^{E} - {\mathbf{\overset{\lower0.5em\hbox{$\smash{\scriptscriptstyle\smile}$}}{a} }}_{k + 1}^{E} } \right)}} > Cv$$

where $${\mathbf{\overset{\lower0.5em\hbox{$\smash{\scriptscriptstyle\smile}$}}{a} }}_{k + 1}^{E}$$ and $${\tilde{\mathbf{a}}}_{k + 1}^{E}$$ denote the best and second best integer solution, respectively. The critical value is often accomplished using empirical results. Many software packages set this value as 3 (Leick 2004; Kroes 2006; Chen et al. 2015). Here, we employed the same value for the ambiguity validation, which worked adequately in our single-frequency case. Note that when the correct ambiguity candidate is resolved, the norm of the noise term of upper triangular system (36) is equal to the squared norm of the ambiguity residuals from LAMBDA, according to the following derivation:

44 $$\begin{aligned} \left\| {{\hat{\mathbf{a}}}_{k + 1}^{E} - {\mathbf{\overset{\lower0.5em\hbox{$\smash{\scriptscriptstyle\smile}$}}{a} }}_{k + 1}^{E} } \right\|_{{{\mathbf{Q}}_{{{\hat{\mathbf{a}}}_{k + 1}^{E} }}^{{}} }} &= \left( {{\hat{\mathbf{a}}}_{k + 1}^{E} - {\mathbf{\overset{\lower0.5em\hbox{$\smash{\scriptscriptstyle\smile}$}}{a} }}_{k + 1}^{E} } \right)^{\text{T}} {\mathbf{S}}_{k + 1}^{\text{T}} {\mathbf{S}}_{k + 1} \left( {{\hat{\mathbf{a}}}_{k + 1}^{E} - {\mathbf{\overset{\lower0.5em\hbox{$\smash{\scriptscriptstyle\smile}$}}{a} }}_{k + 1}^{E} } \right) \hfill \\ & = \left\| {{\mathbf{S}}_{k + 1} {\hat{\mathbf{a}}}_{k + 1}^{E} - {\mathbf{S}}_{k + 1} {\mathbf{\overset{\lower0.5em\hbox{$\smash{\scriptscriptstyle\smile}$}}{a} }}_{k + 1}^{E} } \right\| \hfill \\ & = \left\| {{\hat{\mathbf{w}}}_{k + 1} - {\mathbf{S}}_{k + 1} {\mathbf{\overset{\lower0.5em\hbox{$\smash{\scriptscriptstyle\smile}$}}{a} }}_{k + 1}^{E} } \right\| = \left\| {{\hat{\mathbf{\mu }}}_{k + 1} } \right\| \hfill \\ \end{aligned}$$

Because the noise error term obeys a standard normal distribution with a mean of zero, the residual term of the correct candidate will maintain normality. The Kolmogorov–Smirnov residual test (K–S test) can be used to compare a sample with a reference normal probability distribution. The K–S statistic quantifies the distance between the empirical distribution function of the sample and the cumulative distribution function of the reference distribution (Marsaglia et al. 2003). Here the null hypothesis for the K–S test is that ${\hat{\mathbf{\mu }}}_{k + 1}$ has a normal distribution $N\left( {{\mathbf{0}},{\mathbf{I}}_{n} } \right)$ where *n* is the dimension of ${\hat{\mathbf{\mu }}}_{k + 1}$. The alternative hypothesis is that ${\hat{\mathbf{\mu }}}_{k + 1}$ does not have that distribution. If one cannot reject the null hypothesis, the integer ambiguity vector and the baseline vector may be correct (Chen and Qin 2012). The squared norm of the optimal ambiguity candidate residuals should be checked by a user-defined Chi square threshold as follows:

45 $$\left\| {{\hat{\mathbf{a}}}_{k + 1}^{E} - {\mathbf{\overset{\lower0.5em\hbox{$\smash{\scriptscriptstyle\smile}$}}{a} }}_{k + 1}^{E} } \right\|_{{{\mathbf{Q}}_{{{\hat{\mathbf{a}}}_{k + 1}^{E} }}^{{}} }} < \chi_{\alpha }^{2} \left( {n,0} \right)$$

where *α* is the probability of rejecting the null hypothesis given that it is true. Once the test above is rejected, it indicates that the measurements may not fit the model of the null hypothesis. Possible explanations such as cycle slips and abnormal data or systematic error should be identified. A good overview of the commonly used quality control procedures and testing theory is presented in Teunissen and Kleusberg (1998) and Teunissen (2000).

## Using the structure to improve the model strength with the baseline state prediction

**Fig. 2:**
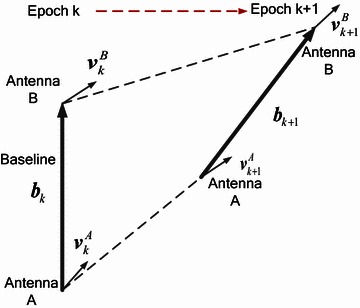
Motion of the baseline

Once the initialization is achieved by validation, all subsequent relative position estimations can be done with the continuous tracking. However, the resolved ambiguity cannot be used for the current baseline estimation if the corresponding satellite is invisible. We designed the algorithm to be efficient and to use the mathematical structure of the recursive estimation to allow the possible integration of other available measurements. For example, in order to improve the success rate under poor satellite visibility, we can integrate the resolved baseline of last epoch into the recursive model. When initialization is achieved, we obtain the first precise estimation of baseline and then predict its value for the next epoch based on the velocity information. For most GPS receivers, accurate position information is provided as well as 3D velocity. As is shown in Fig. 2, the baseline vector ***b*** is defined as a vector from reference antenna A to another antenna B, and the measurements of the velocity vectors of both antennas are given as ${\mathbf{v}}_{k}^{A}$ and ${\mathbf{v}}_{k}^{B}$ at epoch *k*.

Assume that the time span is very short and the accuracy of velocity measurements is sufficiently high, the baseline vector at epoch *k* + 1 can be predicted as follows:

46 $${\tilde{\mathbf{b}}}_{k + 1} = {\mathbf{\overset{\lower0.5em\hbox{$\smash{\scriptscriptstyle\smile}$}}{b} }}_{k} \left( {{\mathbf{\overset{\lower0.5em\hbox{$\smash{\scriptscriptstyle\smile}$}}{a} }}_{k} } \right) + \frac{1}{2}\left[ {\left( {{\mathbf{v}}_{k}^{B} - {\mathbf{v}}_{k}^{A} } \right) + \left( {{\mathbf{v}}_{k + 1}^{B} - {\mathbf{v}}_{k + 1}^{A} } \right)} \right](t_{k + 1} - t_{k} ) + {\mathbf{w}}_{k,k + 1}$$

where $${\mathbf{\overset{\lower0.5em\hbox{$\smash{\scriptscriptstyle\smile}$}}{b} }}_{k} \left( {{\mathbf{\overset{\lower0.5em\hbox{$\smash{\scriptscriptstyle\smile}$}}{a} }}_{k} } \right)$$ is the fixed ambiguity vector at epoch *k* and $${\mathbf{w}}_{k,k + 1}$$ is the measurement noise vector. The variance–covariance matrix can be obtained as

47 $${\mathbf{Q}}_{{{\tilde{\mathbf{b}}},k + 1}} = {\mathbf{Q}}_{{{\mathbf{\overset{\lower0.5em\hbox{$\smash{\scriptscriptstyle\smile}$}}{b} }},k}} + \frac{1}{2}\left[ {\left( {{\mathbf{Q}}_{v,k}^{B} + {\mathbf{Q}}_{v,k}^{A} } \right) + \left( {{\mathbf{Q}}_{v,k + 1}^{B} + {\mathbf{Q}}_{v,k + 1}^{A} } \right)} \right](t_{k + 1} - t_{k} )^{2}$$

So far, if we define ${\mathbf{p}}_{k + 1} \equiv {\mathbf{y}}_{k + 1} - {\mathbf{B}}_{k + 1} {\tilde{\mathbf{b}}}_{k + 1}$, the following equation can be derived from (1)

48 $${\mathbf{p}}_{k + 1} {\mathbf{ = A}}_{k + 1} {\mathbf{a}}_{k + 1} + {\mathbf{u}}_{k}$$

The v–c matrix is written as

49 $${\mathbf{Q}}_{{{\mathbf{p}}_{k + 1} }} = {\mathbf{Q}}_{{{\mathbf{y}}_{k + 1} }} + {\mathbf{B}}_{k + 1}^{{}} {\mathbf{Q}}_{{{\tilde{\mathbf{b}}},k + 1}} {\mathbf{B}}_{k + 1}^{\text{T}}$$

By applying the Cholesky factorization, we decompose the v-c matrix ${\mathbf{Q}}_{{{\mathbf{p}}_{k + 1} }}$ into

50 $${\mathbf{Q}}_{{{\mathbf{p}}_{k + 1} }} = {\mathbf{L}}_{k + 1}^{p} \left( {{\mathbf{L}}_{k + 1}^{p} } \right)^{\text{T}}$$

Multiplying (48) by inverse of ${\mathbf{L}}_{k + 1}^{p}$ from the left and combining it with (36) gives

51 $$\left[ {\begin{array}{*{20}c} {{\hat{\mathbf{w}}}_{k + 1} } \\ {\left( {{\mathbf{L}}_{k + 1}^{p} } \right)^{ - 1} {\mathbf{p}}_{k + 1} } \\ \end{array} } \right] = \left[ {\begin{array}{*{20}c} {{\mathbf{S}}_{k + 1} } \\ \hline {\begin{array}{*{20}c} {\mathbf{O}} &\vline & {\left( {{\mathbf{L}}_{k + 1}^{p} } \right)^{ - 1} {\mathbf{A}}_{k + 1} } \\ \end{array} } \\ \end{array} } \right]{\mathbf{a}}_{k + 1}^{E} + \left[ {\begin{array}{*{20}c} {{\hat{\mathbf{v}}}_{k + 1} } \\ {\left( {{\mathbf{L}}_{k + 1}^{p} } \right)^{ - 1} {\mathbf{u}}_{k} } \\ \end{array} } \right]$$

We are still able to obtain the upper triangular system by applying the orthogonal transformation on both side of (51) in a similar method as used in (35). The approach above demonstrates the flexibility of the recursive model. There are many approaches that use velocity to predict the baseline, such as the state-space models and Kalman filter (Chang and Huang 2005; Wang and Lai 2015). In these approaches, the user motion can be modeled using one of many common dynamic models (random walk, constant velocity, 1st order GM, etc.) while the ambiguities can be modelled as random constants.

## Results of kinematic experiment

**Fig. 3:**
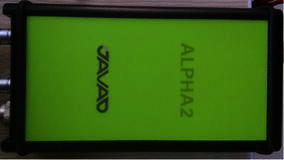
The JAVAD Alpha2 GPS receiver

**Fig. 4:**
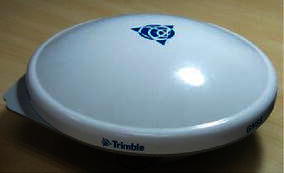
The Trimble^®^ Zephyr™ 2 antenna

The advantage of this proposed method is that the initialization phase can be achieved in motion, since only (11) is utilized to construct the recursive model, which is independent of the baseline status. The success rate of integer ambiguity estimation is also insensitive to satellite setting. To test the actual performance of this extended ambiguity resolution technique with limited satellite visibility, two experiments were performed in urban environments. Both experiments used the receiver onboard the remote control car (telecar) as the rover, shuttling in the street by receiving remote control commands. The reference station was located at a crossroad. A JAVAD Alpha 2 GPS receiver was used, as shown in Fig. 3. Since the advanced multipath reduction techniques are utilized for both carrier phase and code measurements, almost all anomalies and satellite multipath were removed with Alpha 2. GPS antenna design can further reduce the effect of multipath. Here we used two Trimble^®^ Zephyr™ 2 antennas that show excellent performance in multipath mitigation, as shown in Fig. 4.

**Fig. 5:**
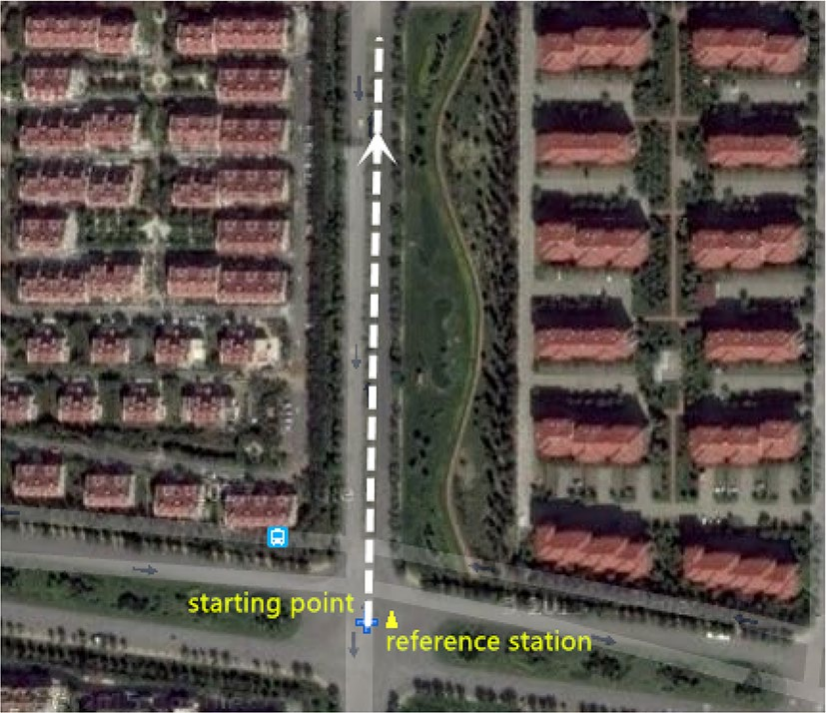
A bird’s eye view of the study area of the first experiment

**Fig. 6:**
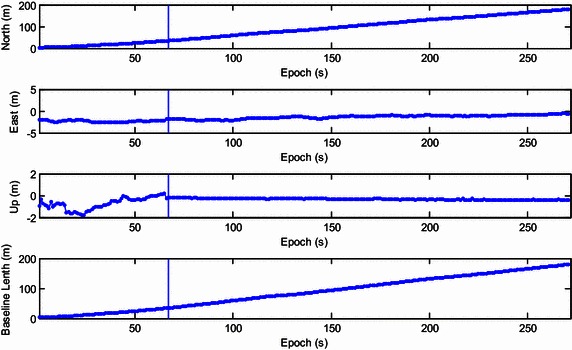
Resolved east, north and up baseline components and baseline length of the first kinematic experiment

For the first experiment, we controlled the telecar moving towards the north. A bird’s eye view of the study area is given in Fig. 5. There are many trees and buildings on both sides of the street, especially in the middle segment. Figure 6 shows the resolved baseline north/east/up components and the baseline length. The ambiguity validation was achieved at epoch 67, marked with the vertical line in Fig. 6. Before this epoch, we estimated the baseline with the float ambiguity vector. After that, the recursive process continued and the optimal ambiguity vector of LAMBDA method was utilized to calculate the baseline. Although the proposed strengthening scheme was not exploited in this experiment, the resolved east, north and up baseline components and baseline length are consistent with the movement of the vehicle. Small fluctuations were found in the east and up components due to rough pavement and inaccurate control.

**Fig. 7:**
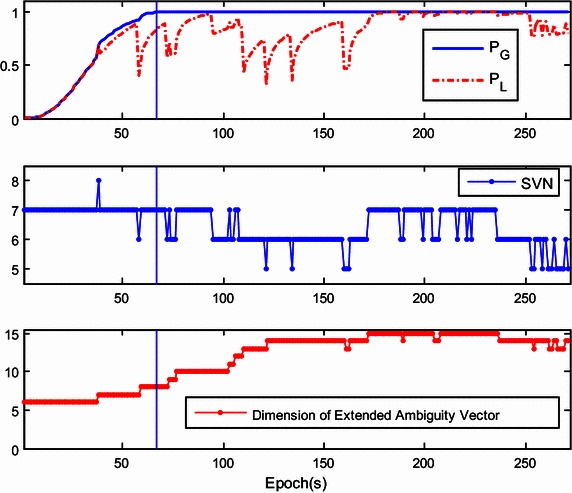
The global/local success rate and the number of visible satellites of the first kinematic experiment

Figure 7 shows the global/local success rate and the number of visible satellites during the first experiment. As is shown, the global success rate was higher than the local success rate and was not sensitive to the drop of the number of visible satellites, even when the number of satellites drops to five. However, unlike the global success rate, the local success rate is very sensitive to the number of visible satellites. When satellite setting occurs, the local success rate drops dramatically. After the rapid drop in success rate, the ‘recovery’ of the local success rate is slow and difficult. This indicates that the estimation of the extended ambiguity vector is more reliable. The lower panel of Fig. 7 shows that the dimension of the extended ambiguity vector increases progressively as satellite rising occurs. Recurrent setting and rising events of satellites will accelerate the growth of the dimension of the extended ambiguity vector. To reduce the necessary computation, the threshold is set as 10 which showed little effect on the global success rate.

**Fig. 8:**
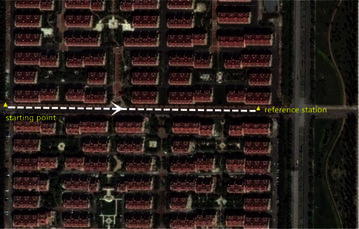
A bird’s eye view of the study area of the second kinematic experiment

**Fig. 9:**
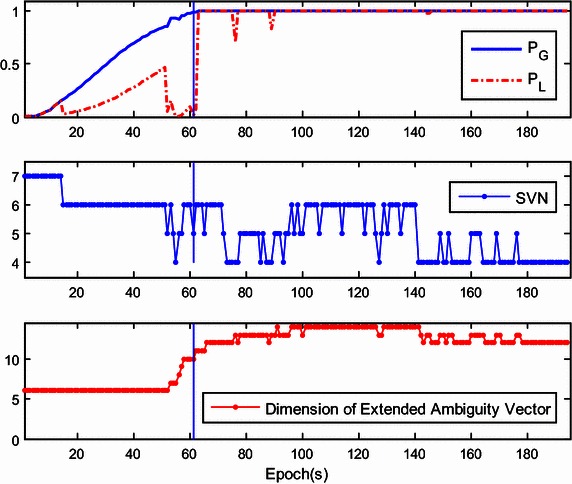
The global/local success rate and the number of visible satellites of the second kinematic experiment

**Fig. 10:**
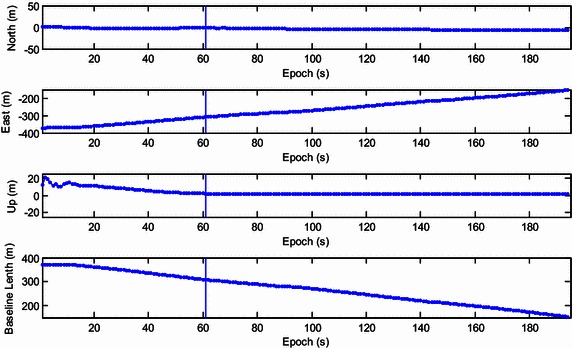
Resolved east, north and up baseline components and baseline length of the second kinematic experiment

In our second experiment, we investigated the actual performance of the proposed method in a more challenging environment. A bird’s eye view of the study area is provided in Fig. 8. The telecar moved towards the east, gradually approaching the reference station. Compared with the first experiment, there were more buildings and trees along the road. Once initialization was achieved, the proposed model-strengthening scheme was exploited during the subsequent epochs. The global/local success rate and the number of visible satellites are shown in Fig. 9, and the vertical line indicates the end of the initialization. The top panel of Fig. 9 shows that the local success rate increased dramatically when the predicted baseline equation was integrated into the recursive algorithm, see (51). This modification made the model more ‘robust’ to satellite setting and the recovery from decline required only a few seconds. The middle panel of Fig. 9 shows the number of visible satellites. Compared with the first experiment, satellite setting and rising was more frequent and the number of visible satellites dropped to 4 many times. However, the global success rate remained close to 1, and was insensitive to the poor satellite visibility. Figure 10 shows the resolved baseline north/east/up components and the baseline length of the second experiment. There was no obvious failure of the baseline estimation, and the inclusion of this estimation in the model allows increased reliability of ambiguity resolution and improved continuity and availability.

## Results of static experiment

**Fig. 11:**
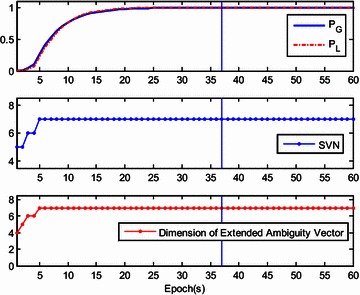
The global/local success rate and the number of visible satellites of the static experiment (from epoch 1–60)

**Fig. 12:**
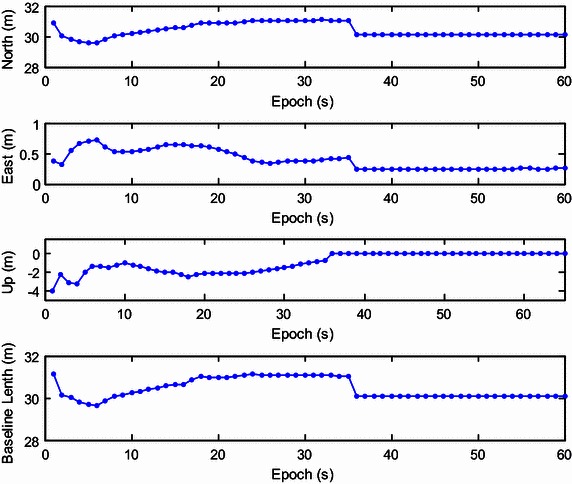
Resolved east, north and up baseline components and baseline length of the static experiment (from epoch 1–60)

**Fig. 13:**
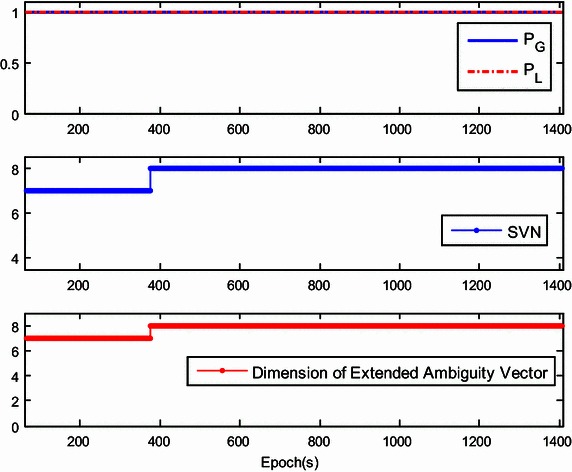
Resolved east, north and up baseline components and baseline length of the static experiment (from epoch 61–1408)

**Fig. 14:**
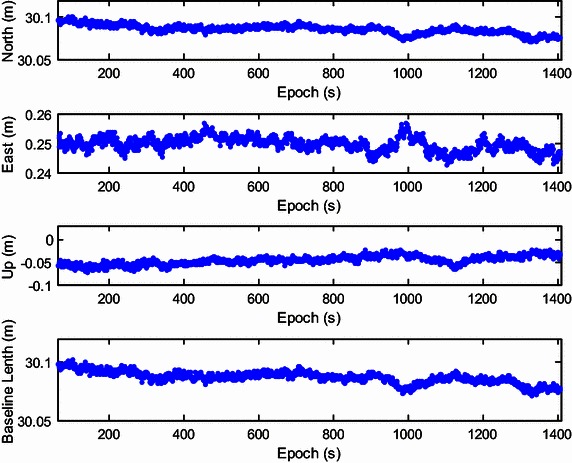
The global/local success rate and the number of visible satellites of the static experiment (from epoch 61–1408)

Validation of corrected position data is not done or possible for the kinematic tests. Thus, we instead used a static test with two JAVAD Alpha 2 receivers to validate the ambiguity-fixed positions by comparison to a known ground-truth position. Data were collected at 1 Hz with a zero cut-off elevation angle for a total of 1408 epochs logged. To obtain a known ground-truth relative position, the distance between the centers of both antennas was measured and the baseline length was approximately 30.08 m. Both antennas were placed on one side of a wide road near the south-north direction. The up component of baseline was close to zero and the north component of baseline was close to the baseline length. The recursive estimation started with four visible satellites (the 3D positions are both fixed for the two antennas), and then the number of tracked satellites grew to seven over several epochs. Initialization was achieved at epoch 37. The results are presented in Figs. 11, 12, 13 and 14, with the data divided into two stages. The first includes epoch 1 to 60, including the initialization phase (Figs. 11, 12), and the second one includes epochs 61 to 1408 (Figs. 13, 14). Figures 11 and 13 show the global/local success rate and the number of visible satellites (the vertical line denotes that the initialization is achieved), and Figs. 12 and 14 show the baseline components and resolved baseline length. With the fixed ambiguities, the average value of north, east, and up measurements of the baseline vector were 30.0857, 0.2497, −0.0466 meters, with standard deviations of 0.0052, 0.0024, 0.0095 meters, respectively, indicating a sub-centimeter level relative position accuracy.

The average of the vertical baseline component was close to zero, but the precision was poorer than the horizontal baseline components because only satellites above the horizon are tracked. The resolved baseline length was very close to the measured value of 30.08 m and the resolved north component was much larger than the east component, consistent with the baseline placement.Thus, the ambiguity-fixed relative position was validated with sub-centimeter level precision.

## Conclusions

A recursive least squares estimator was presented for reliable GPS single frequency kinematic relative positioning in difficult environments. The initialization can be achieved in motion, even if satellite setting and rising events occur frequently during this phase. In order to improve the success rate of ambiguity resolution, an ambiguity dimension expansion method was developed by integrating previous and available information into the current ambiguity estimation equation. The recursive model can be further strengthened using the predicted baseline model or the state-space model. The success rates and relative positioning performance were measured in actual experiments in urban environments. The results reveal that reliable ambiguity estimation can be achieved in motion during initialization. The proposed algorithm also increases the continuity and availability level of relative positioning, even if the number of visible satellites frequently decreases to 4 during the observation.
